# Laparoscopic sleeve gastrectomy as a hybrid day care procedure: a case series of the first 53 patients at a tertiary care center

**DOI:** 10.1007/s13304-023-01591-8

**Published:** 2023-08-06

**Authors:** Mohammad S. Alshahrani, Ayman M. Babiker, Youssuf A. Alsuhaibani

**Affiliations:** 1 Surgical Oncology Department, Consultant Hepatobiliary and Upper GI Surgery, King Fahad Medical City, Riyadh, Saudi Arabia; 2 Surgical Oncology Department, Consultant General Surgery, King Fahad Medical City, Riyadh, Saudi Arabia; 3 Surgical Oncology Department, Consultant Colorectal and Bariatric Surgery, King Fahad Medical City, Riyadh, Saudi Arabia

**Keywords:** Sleeve gastrectomy, Bariatric Surgery, Metabolic Surgery, Enhanced recovery after surgery, Ambulatory bariatric surgery, Day care surgery

## Abstract

Laparoscopic sleeve gastrectomy (LSG) is the most common bariatric procedure. Outpatient LSG is gaining popularity, but the literature is conflicting regarding its safety. Innovative approaches are needed to improve access to bariatric surgery. In this pilot study, we proposed an alternative approach to assess the safety and efficacy of LSG in selected cases as hybrid day care surgery. Data were collected retrospectively from 53 patients who underwent LSG between June 2017 and September 2020 using a hybrid day care approach. Outcomes of the study included patient demographics, clinical characteristics, and outcome variables, including conversion to inpatient care, emergency room visits after discharge, and patient satisfaction. Fifty-three patients (68% females) were included in the study. Mean age was 35.32 years, and mean preoperative body mass index was 42.93. The most common comorbidity was type 2 diabetes mellitus (30.2%), followed by hypertension (15.09%), hypothyroidism (13.2%), and dyslipidemia (9.4%). One (1.89%) patient visited the emergency room because of abdominal pain and was managed and discharged with analgesia. Readmission within 24 h of discharge was not required. One (1.89%) patient developed a staple line gastric leak two weeks after the surgery and was successfully managed with a gastric stent. The series had no mortality, with high patient and family satisfaction. Our approach to performing LSG in hybrid day care surgery is safe and feasible. Adopting this protocol will improve the utilization of resources, while maintaining high levels of patient satisfaction with safety outcomes comparable to the current practice.

**Graphical abstract:**

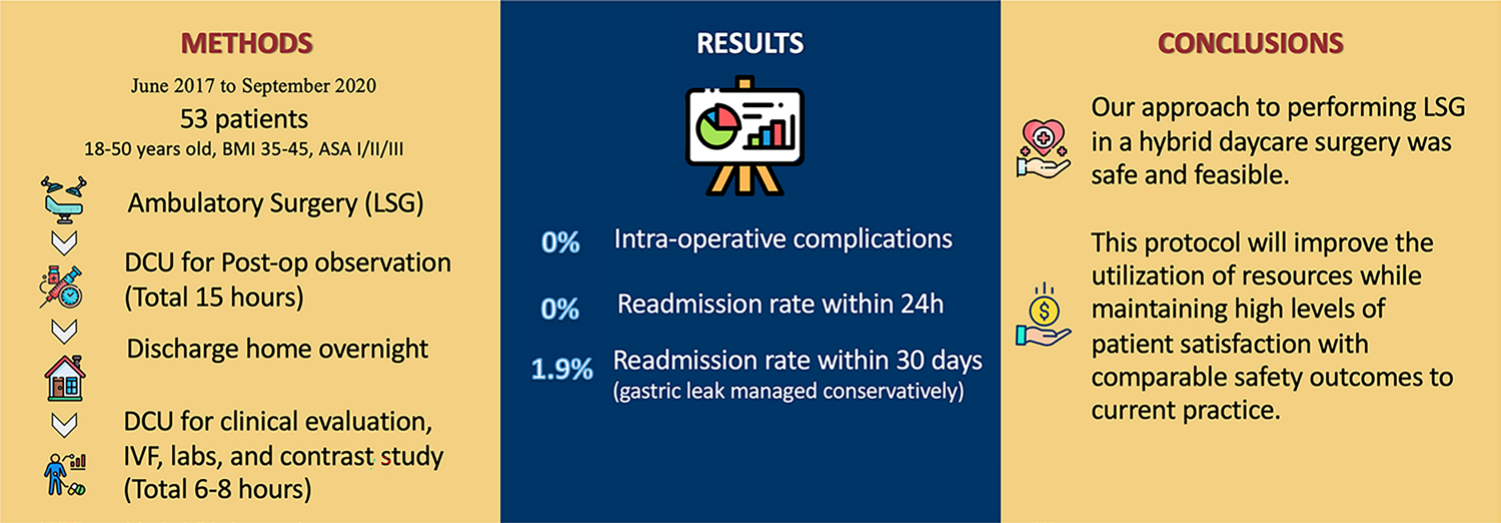

## Introduction

The World Health Organization (WHO) defines obesity as an “abnormal or excessive fat accumulation that may impair health” [1]. Obesity continues to accelerate resulting in an unprecedented epidemic that shows no significant signs of slowing down. Over the past few decades, Saudi Arabia has become increasingly westernized and has one of the highest prevalence rates of obesity and overweight [2]. Obesity in Saudi Arabia is a significant cause of concern, with seven out of ten individuals experiencing this problem [3]. Previous studies related to the prevalence of obesity in the Kingdom of Saudi Arabia indicate an increasing trend in obesity and overweight, which are significant sources of several other diseases, including hypertension, diabetes, obstructive sleep apnea, hyperlipidemia, and osteoarthritis. Bariatric surgery has yielded excellent weight loss results and reduced cardiovascular events [4]. When indicated, laparoscopic sleeve gastrectomy (LSG) is most commonly performed as an inpatient procedure.

Laparoscopic sleeve gastrectomy (LSG) is the most common bariatric surgical procedure worldwide [5]. Several studies have shown that LSG, as an ambulatory procedure, is safe, effective, and feasible in carefully selected patients [6]. Additionally, outpatient bariatric surgery may decrease costs and improve accessibility to bariatric surgery [7].

Day care surgery has proven its efficacy for many procedures, such as hernias and cholecystectomies, and has become the standard pathway. The benefits of this approach include reducing the risks of hospital-acquired infections, improving the quality of care, and achieving high patient satisfaction levels without additional risks. Additionally, a significant decrease in the cost of admission to a day care surgery unit was observed. This approach has also been reported for LSG with promising initial results [8, 9].

In this study, we proposed an alternative approach for handling selected cases that require LSG. This approach involves keeping the patient in a day care unit (DCU) for 15 h on the first day, and then discharging the patient home to be readmitted to the same unit the next day for 6–8 h when clinical care can be completed. We named this approach a hybrid day care procedure. To the best of our knowledge, this has not been reported before. Herein, we report our experience with the first 53 cases to be treated using this approach in a tertiary care center.

## Materials and methods

### Study design, sample, and setting

This study is a retrospective case series of 53 patients over 39 months (between June 1, 2017 and September 30, 2020), and LSG was performed as a hybrid day care procedure. This approach began as an operational project at our institution. We obtained approval from the authorities after multidisciplinary meetings with all services involved in this project. Selection criteria were considered as follows:

- Age 18–50 years.
- Patients living within a 1-h travelling distance from the hospital or a suitable accommodation.
- Body mass index (BMI) of < 45 kg/m^2.^
- No previous upper abdominal operations.
- Controlled diabetes mellitus (HbA1c ≤ 8) and hypertension.
- Absence of symptoms suggesting obstructive sleep apnea, chronic obstructive pulmonary disease, cardiac, or psychiatric illnesses.
- Absence of hiatus hernia or endoscopic evidence of gastroesophageal reflux disease.
- No anti-coagulation medications.
- American Society of Anesthesiologists I, II, or III.
- Patients who need additional procedures like cholecystectomy have been excluded.

Additionally, further social criteria were considered as well:

- Patients/families must be willing to cooperate and comply with postprocedural instructions after receiving adequate information and an opportunity to discuss any anxieties.
- Escort: responsible for patient care and able to accompany them at home and supervise their recovery at home for at least 24 h.
- Transport: Suitable transportation must be available to transport the patient home post-surgery and return to the hospital in case of an emergency.
- Social Support: Patients must have access to telephone services that are readily available at all times.

### Data collection tool

The data collection tool comprised two parts. The first part included patients' demographics and clinical characteristics. The second part consisted of outcome variables, prevalence of intra- and postoperative complications, and readmission rate within 24 h and 30 days, postoperatively.

### Preoperative management

The first step was seeing and evaluating patients in the bariatric surgery clinic after being accepted through our eligibility system from outside hospitals or other hospital departments, such as bariatric medicine. The patients were then clinically evaluated for eligibility for a day care procedure, according to the selection criteria. Next, complete workup investigations were requested, and referrals were arranged to other specialties, such as nutrition, gastroenterology for gastroscopy, and radiology for abdominal ultrasound and chest X-ray. After that, the surgical team saw the patient in the clinic one month before the procedure date to check all workup results. Once the preoperative workup was completed, the surgical team discussed the surgical procedure with the patient (procedure details, preoperative and postoperative instructions, technique, complications, and follow-up plan), allocated operating room time and DCU bed for two consecutive days, and then referred the patient to the pre-anesthesia clinic in agreement with the anesthesia team.

### Day of surgery

The patients were admitted to the DCU at 07:00 and evaluated by the surgical team and nursing staff. The nutritionist met with the patients preoperatively to explain the postoperative plan. The respiratory therapist provided instructions for incentive spirometry before and after the operation. The same bariatric surgeon who has experience with LSG performed all the operations. Abdominal drains were never left in place. Postoperatively, the patients were admitted to the recovery room and then transferred to DCU to assess vital signs, nausea, vomiting, or pain. Oral intake (sips of water) was started 2 h after full recovery from anesthesia. The primary surgical team performed a clinical evaluation at the end of working hours (16:30) for any event that mandated keeping the patient an inpatient. Patients were discharged home if they fulfilled the criteria for discharge (hemodynamic stability, ambulating with normal room air saturation, pain and nausea controlled, and tolerating oral fluids) and were aware of when to visit the ER and to have written instructions and appointments for tomorrow DCU admission and Gastrografin study.

### Day one post-surgery

The patient was admitted into the DCU at 08:00 and then evaluated by the surgical team and nursing staff for pain, nausea, vomiting, hydration status, and hemodynamic stability, and managed accordingly. The patient received intravenous fluids as determined by clinical evaluation. Additionally, an upper gastrointestinal study with oral contrast was performed and evaluated by the primary team before discharge. A nutrition specialist saw all the patients. A follow-up appointment after one week was arranged, and instructions were provided to the patient. The total stay on the second day varied between 6 and 8 h.

### Ethical considerations

Before the study was conducted. Identities of the patients were kept confidential and anonymous. Due to the retrospective nature of the study, informed consent was not obtained.
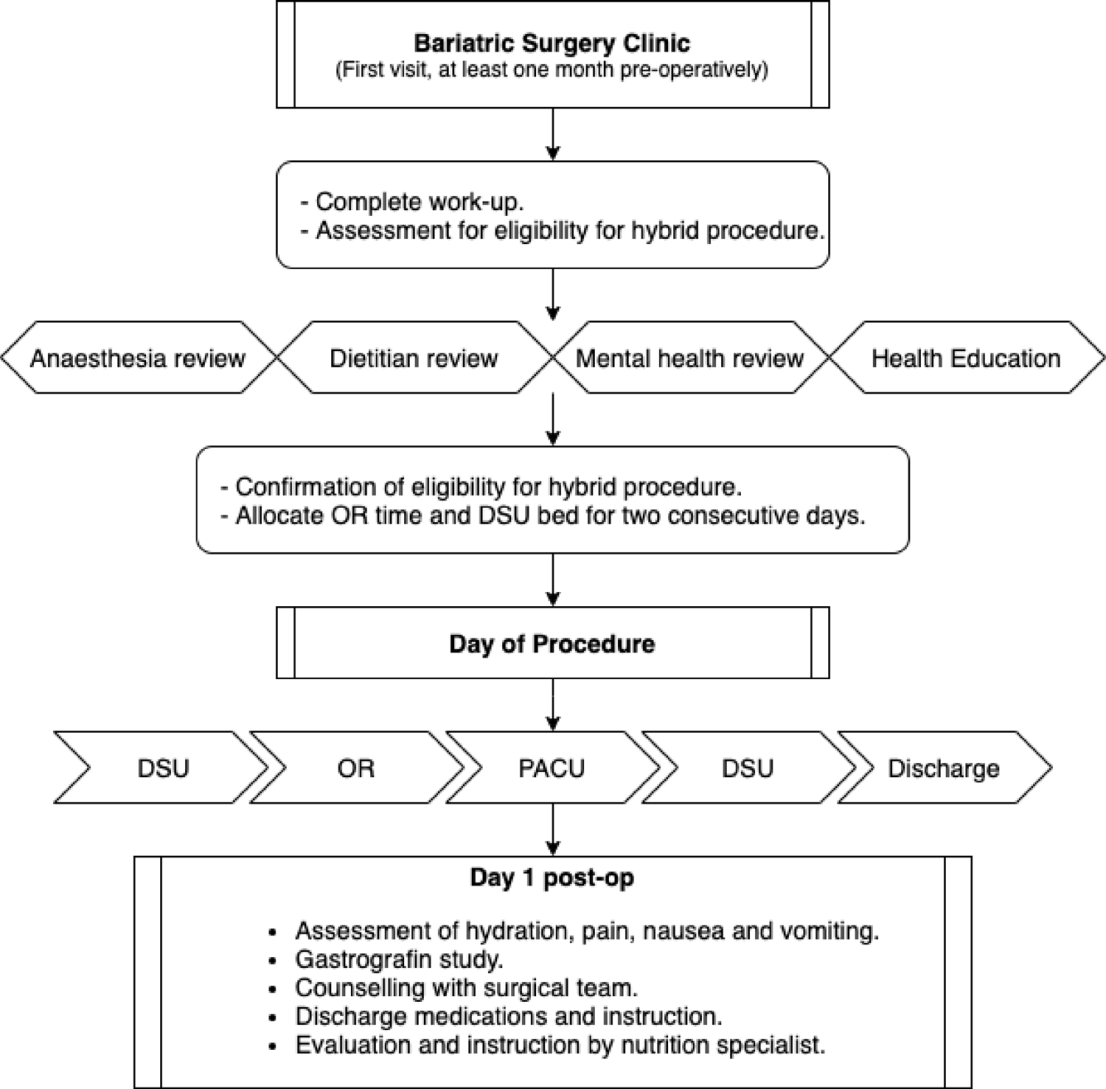


## Results

A total of 53 patients underwent LSG during the study period (table 1). Thirty-six patients were females (68%), and their mean age was 35.32 years. The BMI ranged between 35 and 45 kg/m^2^ with a mean of 42.27 ± 2.78 kg/m^2^. In terms of comorbidities, 16 patients had type II diabetes mellitus (30.2%) and eight had hypertension (15%). Other comorbidities included hypothyroidism (13.2%), dyslipidemia (9.4%), bronchial asthma (5.6%), and non-complicated gallstone disease (5.6%). There were no intraoperative complications among all the patients, and only one (1.89%) patient, the first case, visited the ER because of pain and was managed with analgesia and then discharged. Readmission within 24 h of discharge was not required. One (1.89%) patient developed a staple line leak two weeks after the surgery and was managed successfully with a gastric stent. No other patient readmission was needed in the 30 days following the procedure Table 2.

**Table 1:** Baseline characteristics of study participants

Age	Mean 35.32 (± 9.15)
BMI	Mean 42.27 (± 2.78)
Female	36 (68%)
Comorbidities	
▪ DM	16 (30.2%)
▪ HTN	8 (15%)
▪ Hypothyroidism	7 (13.2%)
▪ Dyslipidemia	5 (9.4%)
▪ Bronchial asthm	3 (5.6%)
▪ Gallstones	3 (5.6%)
ASA	
▪ 1	0 (0%)
▪ 2	44 (83%)
▪ 3	9 (17%)
▪ 4	0 (0%)
▪ 5	0 (0%)
OR Time	Mean 64.17 min (± 9)
EBL	Mean 25.85 ml (± 15.75)

**Table 2:** Outcomes

Intra-operative complications	0 %
Conversion to an open approach	0
ER visit within 24 h	
▪ Pain	1 (1.89)
Post-operative complications	
▪ Leak	1 (1.89)
▪ Bleeding	0
Reoperation within 30 days	0
Readmission within 24 h	0
Readmission within 30 days	1.89

## Discussion

The results of this pilot study confirmed the safety and feasibility of this approach. Our results are comparable to those reported in the literature with ambulatory bariatric surgery having lower readmission rates. Propensity score–matched analysis of the Metabolic and Bariatric Surgery Accreditation and Quality Improvement Program Registry (MBSAQIP) comparing same-day discharge following LSG to inpatient management showed a similar risk of a leak (0.56% versus 0.40%; relative risk [RR], 1.419; 95% CI 0.896–2.245; P 5.133), bleeding (0.38% versus 0.31%; RR, 1.250; 95% CI 0.731–2.138; P 5.414), 30-day reoperation (0.81% versus 0.56%; RR, 1.432; 95% CI 0.975–2.104; P 5.066), and 30-day morbidity (1.15% versus 1.01%; RR, 1.139; 95% CI 0.842–1.541; P 5.397) [10]. However, patients discharged on the same day had a significantly increased risk of 30-day readmission (3.35% vs. 2.79%; RR, 1.202; 95% CI 1.009–1.432; P 5.039). Other reports have also shown that unplanned hospital readmission following outpatient LSG occurs in approximately 8–10% of patients [6, 11]. In the study by Ignat et al., day care LSG decreased the cost by 14% but 46.6% of the study patients had an unplanned event. 7 overnight stays (23.3%), 3 readmissions (10%), and 4 unscheduled consultations (13.3%). Most unplanned emergency department visits and admissions are secondary to nausea and vomiting [12]. An inability to tolerate oral intake could be mitigated by planned reevaluation and hydration the following day, as in our protocol. In our series, none of the patients required readmission within the first 24 h, and only 1.89% of the patients were readmitted within 30 days. Leak rates following LSG remain at approximately 2.4–3.3% [13, 14]. Our leak rate of 1.89% is consistent with those reported in other studies.

Chadha etal described a similar approach using a “hybrid care hotel” model in which patients perioperatively were managed at a hotel with daytime nursing care and nighttime virtual registered nurse monitors. A variety of procedures were included and patient satisfaction was reported through a questionnaire. Of the 56% who responded 94% reported satisfaction and would recommend the programme to other patients. However the study did not analyze outcomes and costs [15].

The concerns of performing LSG in a DCU for a maximum of 23 h are persistent nausea, poor pain control, dehydration, and insufficient monitoring for the early detection of serious complications. These concerns could be minimized by dividing the DCU admission into two consecutive days (15 and 6–8 h, respectively) for a more extended period of observation and rehydration. The patient could be given discharge instructions by the same surgical team and nutrition specialist at a more convenient time, while the patient is fully awake and oriented. Furthermore, this approach eliminates the need for a night nursing shift and reduces the demand for the on-call team. Careful patient selection with preoperative screening and the exclusion of patients with severe comorbidities from the outpatient setting will significantly reduce the risk of these complications. Observing patients on the second day after surgery to assess nausea, pain control, and hydration status may play an essential role in the feasibility of performing LSG in DCU. This approach can also improve the quality of care by ensuring that patients are seen by the same staff and managed using consistent protocols. Consistency among surgical teams has been shown to affect efficiency and patient outcomes [16].

This case series of our first 53 obese human patients showed that this approach is feasible and has no safety concerns. These findings led to the adoption of this protocol in our institution for LSG fulfilling the criteria, and also generated broad interest in applying the concept to other procedures.

We hypothesize these changes may possible reduce strain on nursing, decrease costs and improve patient satisfaction but would require further studies to validate these claims.

## Conclusion

Hybrid day care LSG is feasible and safe if patients are carefully and correctly selected. The morbidity and mortality rates in our pilot study were comparable to those in conventional management and may provide an excellent alternative to the current day surgical approach. Proper assessment and management of vital signs, pain, nausea, vomiting, and hydration are the cornerstones of successful programs.
